# Changes in cerebral autoregulation and vasoreactivity after surgical aortic valve replacement: a prospective study

**DOI:** 10.1113/EP090502

**Published:** 2022-11-20

**Authors:** Tiago Pedro, Andreia Costa, Juliana Ferreira, Ana Luísa Rocha, Elson Salgueiro, Gilberto Pereira, Elsa Azevedo, Pedro Castro

**Affiliations:** ^1^ Department of Neuroradiology Centro Hospitalar Universitário de São João, E.P.E. Porto Portugal; ^2^ Department of Neurology Centro Hospitalar Universitário de São João, E.P.E. Porto Portugal; ^3^ Department of Clinical Neurosciences and Mental Health Faculty of Medicine of University of Porto Porto Portugal; ^4^ Cardiovascular Research and Development Unit Faculty of Medicine of University of Porto Porto Portugal; ^5^ Department of Cardiothoracic Surgery Centro Hospitalar Universitário de São João, E.P.E Porto Portugal; ^6^ Department of Physiology and Cardiothoracic Surgery Faculty of Medicine of University of Porto Porto Portugal

**Keywords:** aortic valve stenosis, cerebral autoregulation, transcranial Doppler ultrasound

## Abstract

Surgical aortic valve replacement (SAVR) alters the natural course of severe aortic stenosis (AS). In this study, we aimed to determine the effects of the disease on dynamic cerebral autoregulation and vasoreactivity (VR) and to assess their changes after SAVR. We recruited 23 patients diagnosed with severe AS eligible for SAVR and 15 healthy matched controls. AS patients had lower mean VR to CO_2_ (*P* = 0.005) than controls, but dynamic cerebral autoregulation was preserved. Cerebral haemodynamics showed no significant change after SAVR. Patients with smaller baseline aortic valve areas presented with smaller low frequency phase changes after surgery (*P* = 0.016). Severe AS does not seem to impact dynamic cerebral autoregulation but does reduce VR to CO_2_. SAVR does not alter cerebral autoregulation nor vasoreactivity.

## Abstract (highlights)

- **What is the central question of this study?**
  How are dynamic cerebral autoregulation and brain vasoreactivity influenced by severe aortic stenosis and its surgical treatment?
- **What are the main findings and their importance?**
  Dynamic cerebral autoregulation is preserved in the long term in patients with severe aortic stenosis and does not change after surgical aortic valve replacement. However, carbon dioxide vasoreactivity is impaired in these patients.

## 1 INTRODUCTION

Aortic stenosis (AS) is the most prevalent primary valvular heart disease across Europe and North America and a common cause of chronic heart failure (CHF). The role of CHF in cerebral haemodynamics has been poorly studied over the years and results on dynamic cerebral autoregulation (dCA) are controversial. dCA maintains cerebral blood flow (CBF) relatively constant regardless of changes in mean arterial pressure (MAP), although recent evidence has shown that this happens particularly within a strict range of MAP and that this relationship is more efficient when MAP is increased (Brassard et al., 2021). On the other hand, vasoreactivity (VR) is the ability of the cerebral arterioles to respond to vasoactive stimuli, namely, hypoxia and hypercapnia. Patients with ischaemic CHF were found to exhibit reduced end‐tidal CO_2_ VR and autoregulation index values compared to healthy controls, possibly due to increased vasoconstriction secondary to a chronic reduction in cardiac output and brain hypoxia (Caldas et al., 2017). Given the poor prognosis of AS if left untreated (Thaden et al., 2014), early intervention is recommended in patients with severe symptomatic AS with reasonable life expectancy using either transcatheter aortic valve implantation or surgical aortic valve replacement (SAVR). In this study, we aimed to assess the changes in dCA and VR to CO_2_ parameters caused by AS and after SAVR.

## 2 METHODS

### 2.1 Ethics approval

The study protocol was conceived in agreement with the *Declaration of Helsinki* with the exception of registration in a database and was approved by the local institutional Ethical Committee (Authorization Number CE15‐15). Before testing, all experimental procedures were explained to participants and written consent was provided.

### 2.2 Participants and study design

This was a prospective study that included 23 patients aged ≥18 years followed between 2018 and 2020 and diagnosed with symptomatic severe aortic stenosis eligible for surgical replacement of the aortic valve. Baseline features were compared against 15 health controls matched for age and sex. Severe aortic stenosis was defined as an aortic‐valve area of lower than 1.0 cm^2^, a mean aortic‐valve gradient of 40 mmHg or more, or a peak aortic‐jet velocity of 4.0 m/s or more. The diagnosis was confirmed by a heart team consisting of at least an imaging cardiologist, an interventional cardiologist and a cardiac surgeon. All patients had symptoms of either New York Heart Association functional class II or higher symptoms of heart failure, syncope, exertional dyspnoea, angina or presyncope by history or on exercise testing. Exclusion criteria were as follows: (1) inability to fully understand and/or give written informed consent for the study protocol; (2) history of cardiac surgery, myocardial infarction, end‐stage renal failure, pulmonary failure, transient ischaemic stroke and stroke within the previous year; (3) symptomatic significant carotid stenosis; (4) presence of DSM‐V criteria for dementia or pseudodementia; and (5) inflammatory or malignant concomitant disease.

### 2.3 Assessments and data acquisition

Demographic (sex and age), echocardiographic (aortic valve area, aortic mean gradient and ejection fraction) and clinical variables (comorbidities and chronic medications) were collected at baseline from medical records. On the day of surgery, patients were evaluated by an experienced neurologist. Participants then underwent transcranial Doppler ultrasound (Doppler BoxX, DWL, Singen, Germany) performed by an experienced neurosonologist. They were asked to refrain from caffeine or alcohol consumption, physical exercise and vasoactive drugs for at least 12 h before the monitoring. The M1 segment of the middle cerebral artery (MCA) was insonated bilaterally to a depth of 45–55 mm with 2‐MHz monitoring probes secured with a headframe and cerebral blood flow velocity (CBFV) was recorded. Beat‐by‐beat heart rate and blood pressure were recorded simultaneously using an electrocardiogram and a Finometer (Finapres Measurement Systems, Arnhem, Netherlands). End‐tidal CO_2_ ($ET_{CO_{2}}$) was measured by non‐invasive capnography (Respsense Nonin, Amsterdam, Netherlands) attached to a nasal cannula.

**FIGURE 1:**
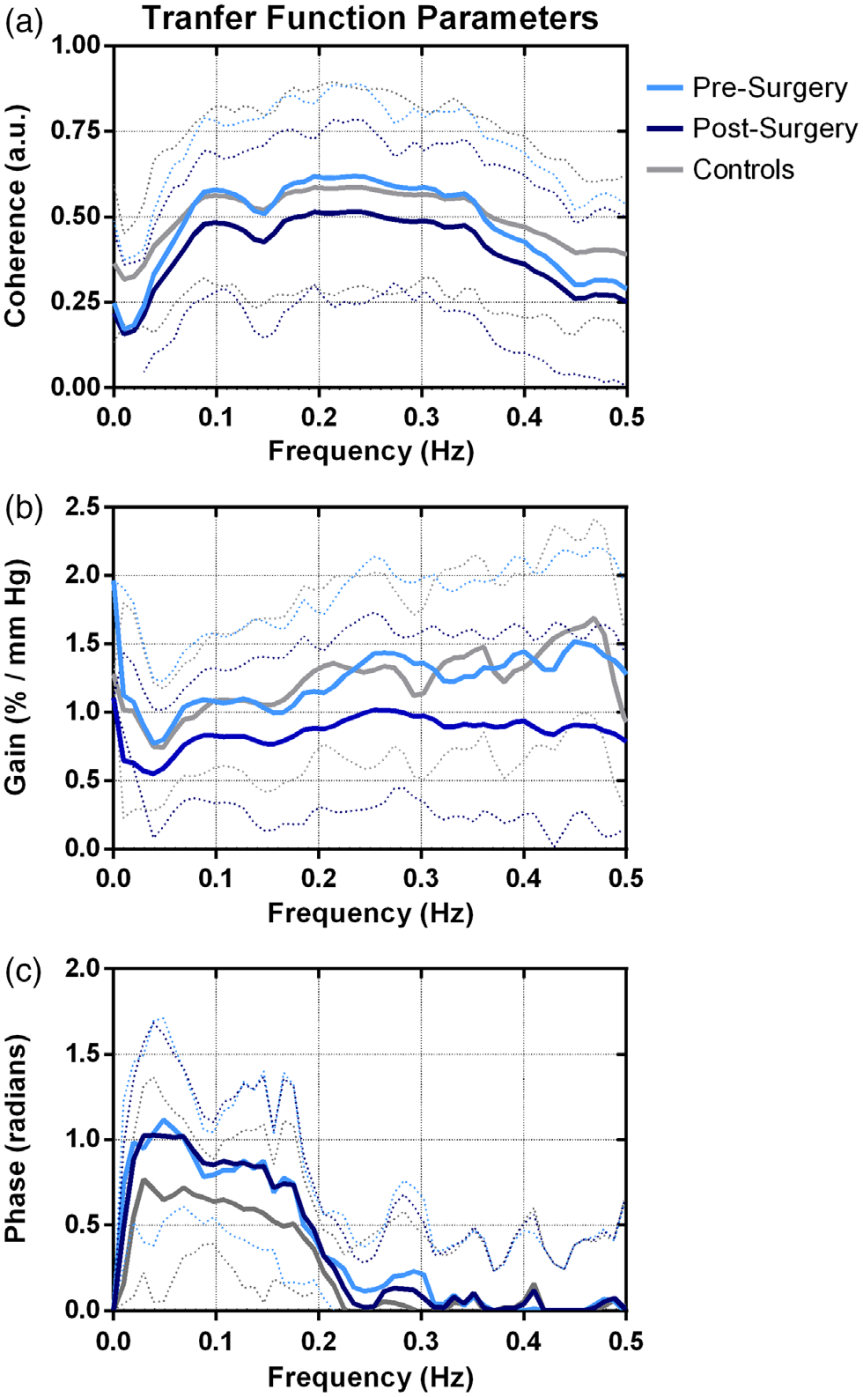
Averaged transfer function parameters for pre‐surgery, post‐surgery, and control patients. (a) Coherence; (b) gain; (c) phase. Dotted lines indicate standard deviation

**FIGURE 2:**
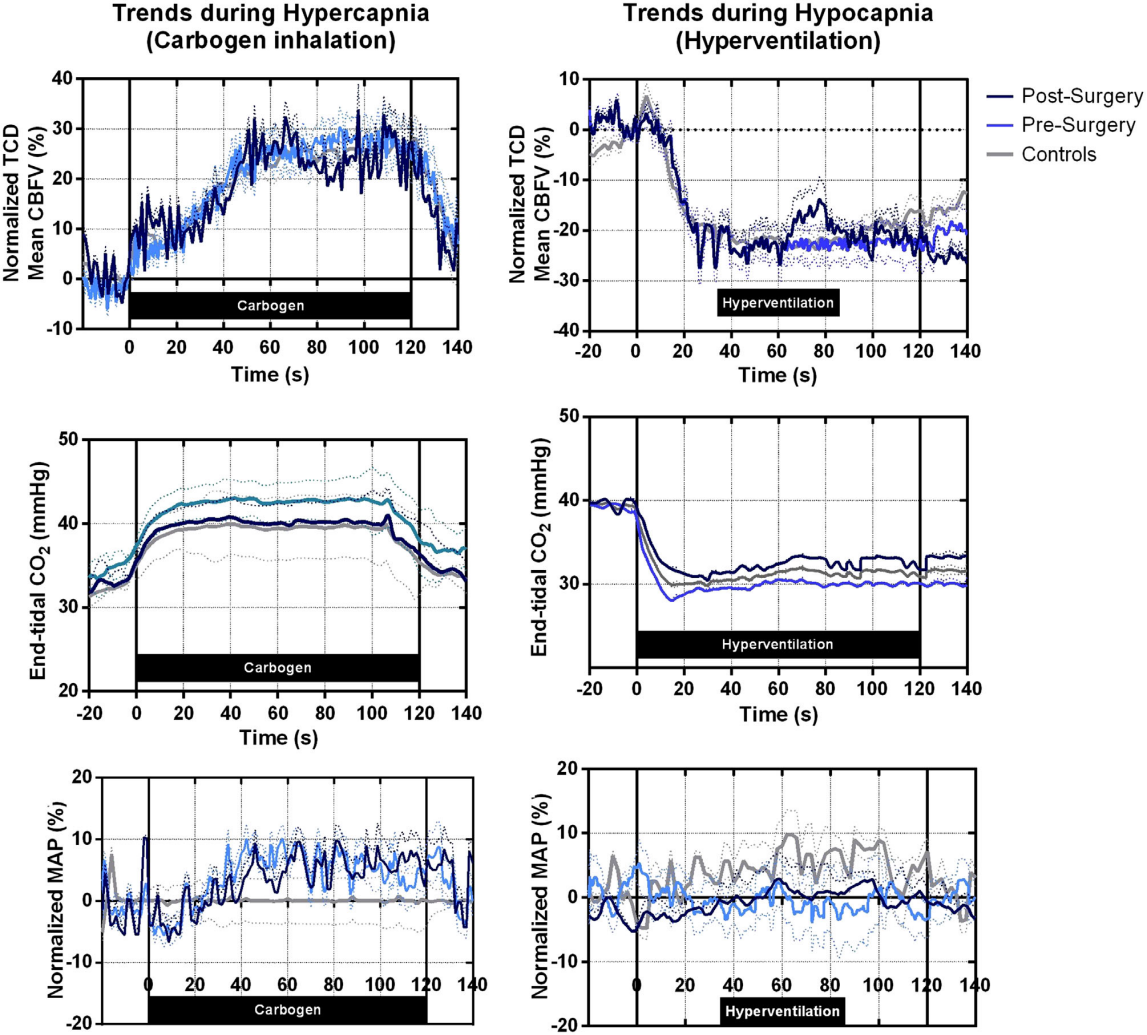
Mean cerebral blood flow velocity (CBFV), end‐tidal CO_2_ and normalized mean arterial pressure (MAP) during hypercapnia and hypocapnia states for pre‐surgery, post‐surgery and control patients. Dotted lines indicate standard deviation. TCD, transcranial Doppler

Data were synchronized offline using MATLAB (The Mathworks, Natick, MA, USA). Cerebral autoregulation was assessed by transfer function analysis accordingly to proposed standards (Panerai et al., 2022) and coherence, gain and phase values were reported at very low (0.02–0.05 Hz), low (0.05–0.20 Hz) and high (0.2–0.5 Hz) frequency bands. The average of the values measured on both sides was reported (Figure 1). Afterward, a vasoreactivity test for CO_2_ was performed. Mean CBFV was measured in the supine position, at rest, in ambient air, during a CO_2_ challenge (inhalation of a 5% CO_2_ + 95% O_2_ mixture for 2 min), and then after 2 min of mild hyperventilation. A step‐change in $ET_{CO_{2}}$ of 7–10 mmHg was guaranteed for hyper‐ or hypocapnia. VR was determined as the slope of the relationship between $ET_{CO_{2}}$ plotted against the mean CBFV at the last 30 s of hyper‐ or hypercapnia and expressed as a change in cerebral blood flow per mmHg change in $ET_{CO_{2}}$. Cerebral VR was determined during hypercapnic and hypocapnic states (Figure 2).

**FIGURE 3:**
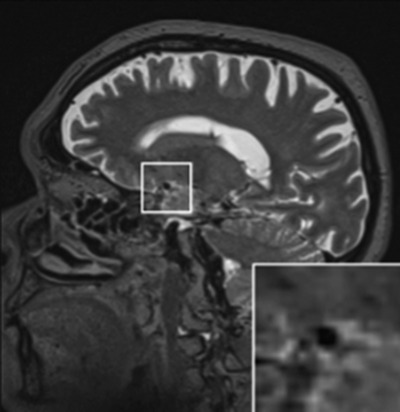
Example of a T2‐weighted MRI sagittal scan of the middle cerebral artery (MCA). The white box indicates the zoomed area showing the lumen of the M1 portion of the MCA

Since studies on dCA using transcranial Doppler rely on the assumption that the MCA diameter is consistent throughout the monitoring, a brain magnetic resonance imaging (MRI) scan was obtained before and after SAVR. Sequences included axial T1‐weighted, axial T2‐weighted, axial diffusion‐weighted imaging, and fluid‐attenuated inversion recovery, collected in a 1.5 tesla MRI scanner (Magnetom Aera, Siemens Healthineers, Erlangen, Germany). The M1 portion of the MCA was identified on orthogonally reconstructed axial three‐dimensional T2‐weighted scans and the greatest and lowest diameters were averaged for each patient (Figure 3). To assess the existence of surgery‐related changes in dCA and VR to CO_2_, the procedures above mentioned were repeated 6 months after SAVR in 12 patients. The remaining patients were lost during follow‐up.

### 2.4 Statistical analyses

Descriptive statistics included mean and standard deviation for continuous variables and absolute and relative frequency for categorical variables. The cerebrovascular conductance index (CVCi) was calculated as middle cerebral artery velocity (MCAv)/MAP to minimize the changes in MAP on cerebral perfusion. Changes in cerebral autoregulation parameters were determined by subtracting post‐operative parameters from baseline values. A comparison of baseline cerebral autoregulation parameters between patients with AS and controls was tested for significance using the Mann–Whitney test. The Wilcoxon signed rank test compared paired changes in autoregulation parameters and vasoreactivity before and after SAVR. The relation between the changes in cerebral autoregulation parameters and aortic stenosis echocardiographic features was assessed with Spearman's correlation coefficient. All statistical analysis was performed using the SPSS Statistics version 26 (IBM Corp., Armonk, NY, USA). A *P*‐value <0.05 (two‐tailed) was considered statistically significant.

## 3 RESULTS

We recruited 23 patients diagnosed with severe AS with a mean age of 74.9 ± 8.1 years and 15 healthy matched controls, eight males and seven females, with a mean age of 75.1 ± 4.3 years. The distribution of age (*P* = 0.286) and sex (*P* = 0.646) did not differ between groups. Baseline demographic and disease characteristics of AS patients are shown in Table 1.

**TABLE 1:** Baseline features of 23 patients eligible for aortic valve replacement surgery

Variables	
Sex, *n* (%)	
Female	9 (39.1)
Male	14 (60.9)
Age (years), mean ± SD	76.2 ± 7.6
Educational level (years)	3.9 ± 3.3
Echocardiographic features, mean ± SD	
Aortic valve area (cm^2^)	0.7 ± 0.2
Aortic mean gradient (mmHg)	49.3 ± 12.0
Ejection fraction (%)	55.0 ± 15.5
Comorbidities, *n* (%)	
Hypertension	17 (73.9)
Dyslipidaemia	17 (73.9)
Diabetes mellitus	8 (34.8)
Ischaemic heart disease	2 (8.7)
Ischaemic stroke	2 (8.7)
Atrial fibrillation	2 (8.7)
Chronic medications, *n* (%)	
ACEI/ARB	5 (21.7)
Calcium channel blockers	3 (13.0)
Diuretics	3 (13.0)
Statins	3 (13.0)
Antiplatelet drugs	3 (13.0)
β‐Blockers	2 (8.7)

Abbreviations: ACE, angiotensin‐converting‐enzyme inhibitors; ARB, angiotensin‐II receptor blockers.

The comparison between pre‐operative dCA and VR parameters between groups and the pairwise changes within AS patients after SAVR are analysed in Table 2. The MFV, coherence, gain and phase were unaffected by SAVR, and the mean MCA diameter remained almost unchanged after SAVR (*P* = 0.674) in the eight patients that repeated brain MRI. Overall brain VR to CO_2_ also did not benefit from the intervention (*P* = 0.463). However, it was significantly lower in non‐surgically treated AS patients compared to healthy controls (*P* = 0.005), albeit subgroup analysis showed this difference was limited to the hypercapnic state (*P* = 0.032). CBFV increased significantly less in patients with severe AS during the hypercapnic state (*P* = 0.022), but no changes during the hypocapnia procedures were found. Aortic stenosis echocardiographic features were correlated with changes in dCA and VR parameters (Table 3). Patients with smaller baseline aortic valve area presented with significantly lower changes of LF phase after SAVR (*p* = 0.016). Changes in the MFV and remaining dCA and VR parameters were not significantly correlated with the aortic valve area nor the mean aortic gradient before surgery.

**TABLE 2:** Comparison of cerebral autoregulation, vasoreactivity to CO_2_, and clinical parameters in the control group and patients with aortic valve stenosis before and after aortic valve replacement surgery

		Patients submitted to aortic valve replacement surgery		*P*	
Parameter, mean ± SD	Control group (*n* = 15)	Before surgery (*n* = 23)	After surgery (*n* = 12)	Baseline group comparison^a^	Pairwise comparison^b^
MFV (cm/s)	57.0 ± 8.9	53.5 ± 15.7	53.9 ± 12.3	0.237	0.583
Mean blood pressure (mmHg)	81.52 ± 13.56	64.10 ± 11.78	70.85 ± 16.28	**0.001**	0.050
CVCi (cm/s/mmHg^2^)	0.71 ± 0.14	0.85 ± 0.23	0.79 ± 0.24	0.112	0.695
$ET_{CO_{2}}$ (mmHg)	37.28 ± 3.34	35.13 ± 3.85	33.12 ± 4.79	0.077	>0.999
MCA diameter (mm)	—	3.26 ± 0.38	3.29 ± 0.36	—	0.674
Heart rate (bpm)	65.34 ± 8.80	65.01 ± 11.58	67.52 ± 12.85	0.843	0.814
Cerebral autoregulation					
VLF coherence	0.44 ± 0.15	0.41 ± 0.17	0.45 ± 0.25	0.636	0.182
LF coherence	0.57 ± 0.16	0.47 ± 0.24	0.49 ± 0.26	0.249	0.638
HF coherence	0.55 ± 0.14	0.51 ± 0.28	0.64 ± 0.27	0.614	0.136
VLF gain (%/mmHg)	0.80 ± 0.34	1.33 ± 1.40	0.86 ± 0.58	0.181	0.308
LF gain (%/mmHg)	1.14 ± 0.33	2.32 ± 3.95	1.10 ± 0.70	0.891	0.480
HF gain (%/mmHg)	1.52 ± 0.44	2.04 ± 2.58	1.53 ± 0.62	0.417	0.638
VLF phase (radians)	0.90 ± 0.31	1.11 ± 0.61	0.97 ± 0.67	0.279	0.272
LF phase (radians)	0.68 ± 0.21	0.79 ± 0.60	0.85 ± 0.52	0.748	>0.999
HF Phase (radians)	0.01 ± 0.25	0.04 ± 0.38	0.01 ± 0.17	0.772	0.875
VR to CO_2_ (%/mmHg CO_2_)					
Overall	4.71 ± 0.97	3.41 ± 1.97	2.18 ± 1.10	**0.005**	0.463
Hypercapnia state	5.6 ± 1.8	3.77 ± 1.91	3.25	**0.032**	0.463
CBFV (%)	51.23 ± 8.51	27.39 ± 20.55	24.83 ± 12.63	**0.022**	0.309
MAP (%)	13.27 ± 14.57	8.27 ± 19.37	9.41 ± 15.57	0.145	0.877
CVCi (%)	21.11 ± 12.06	11.11 ± 14.06	5.98 ± 13.67	**0.008**	0.577
Hypocapnia state	1.7 ± 0.4	2.9 ± 2.1	2.77 ± 0.99	0.081	0.921
CBFV (%)	−23.54 ± 12.71	−19.51 ± 10.24	−15.46 ± 12.6	0.432	0.885
MAP (%)	−4.46 ± 11.39	−2.51 ± 15.4	−1.34 ± 14.9	0.562	0.158
CVCi (%)	−15.23 ± 11.34	−12.23 ± 15.34	−10 ± 12.61	**0.030**	0.125

Very‐low (VLF 0.02–0.05 Hz), low (LF 0.05–0.2 Hz) and high (HF 0.2–0.5 Hz) frequency bands.

^a^ *P* < 0.05 significance value for differences in the pre‐operative autoregulation and vasoreactivity parameters between patients with aortic valve stenosis and a control group obtained with the Mann–Whitney test.

^b^ *P* < 0.05 significance value for differences between before and after aortic valve replacement surgery obtained with the Wilcoxon test. *P*‐values in bold indicate statistical significance. Abbreviations: CBFV, cerebral blood flow velocity; CVCi, cerebrovascular conductance index; $ET_{CO_{2}}$, end tidal CO_2_; HR, heart rate; MAP, mean arterial pressure; MCA, middle cerebral artery; MFV, mean flow velocity; VR, vasoreactivity.

**TABLE 3:** Overall changes from baseline in cerebral autoregulation, vasoreactivity to CO_2_ and clinical parameters after aortic valve replacement surgery and their relationship with disease features

	Aortic valve area		Mean aortic gradient	
Parameter	Spearman's rho	*P*	Spearman's rho	*P*
MFV	−0.379	0.402	0.352	0.353
Mean blood pressure	−0.703	0.078	−0.318	0.404
CVCi	−0.162	0.728	0.402	0.284
Mean heart rate	0.180	0.699	−0.268	0.486
Cerebral autoregulation				
VLF coherence	−0.126	0.788	0.360	0.342
LF coherence	−0.703	0.078	0.184	0.635
HF coherence	−0.252	0.585	0.276	0.472
VLF gain	−0.324	0.478	0.351	0.354
LF gain	−0.324	0.478	0.427	0.252
HF gain	−0.523	0.229	0.444	0.232
VLF phase	0.541	0.210	0.544	0.130
LF phase	0.847	0.016*	0.100	0.797
HF phase	0.378	0.403	0.084	0.831
$ET_{CO_{2}}$ (%)	−0.306	0.504	0.176	0.651
VR to CO_2_	−0.521	0.150	−0.432	0.213

Very‐low (VLF 0.02–0.05 Hz), low (LF 0.05–0.2 Hz) and high (HF 0.2–0.5 Hz) frequency bands.

* *P* < 0.05 significance value for correlation between autoregulation and vasoreactivity parameters and echocardiographic disease features obtained with Spearman's correlation coefficient. Abbreviations: CVCi, cerebrovascular conductance index; $ET_{CO_{2}}$, end tidal CO_2_; HR, heart rate; MFV, mean flow velocity; VR, vasoreactivity.

Mean blood pressure was lower in AS patients compared to healthy controls (*P* = 0.001) but increased significantly after surgery. Adaptations in vascular conductance were also significantly compromised in both hypercapnic (*P* = 0.008) and hypocapnic states (*P* = 0.030), although the steady‐state CVCi was preserved in AS patients at baseline (*P* = 0.112).

## 4 DISCUSSION

To our best knowledge, we are the first to study the changes in dCA and VR after SAVR in patients with severe AS. Our main findings are (1) dCA parameters in patients with severe AS did not differ from healthy matched controls; (2) cerebral vasoreactivity (CVR) was significantly impaired in AS patients; and (3) SAVR does not improve cerebral autoregulation or vasoreactivity in AS patients.

Systemic arterial hypertension (SAH) is a common pitfall of AS that was present in most of our patients and is responsible for inducing hypertrophy and remodelling of the vessel wall that in the chronic phase contributes to increasing cerebrovascular resistance. Although our AS cohort showed a significantly lower mean arterial blood pressure when compared to matched controls, the majority of patients had been previously diagnosed with SAH. Interestingly, different studies have shown that hypertensive individuals maintain their ability to autoregulate the CBF due to greater vascular distensibility instead of muscular activity, which may explain the lack of significant impairment in dCA in our AS cohort (Machado et al., 2020; Serrador et al., 2005). Moreover, the lower CVR found in AS patients may be also due to chronically elevated blood pressure as previously documented. Possible mechanisms behind this finding include the decrease in endothelial levels of nitric oxide, secondary stroke and white matter lesions, as well as anatomical changes in the arterial wall. The micro‐ and macrovascular brain damage secondary to hypertension is also associated with lower vasoreactivity (Hajjar et al., 2010).

Another important aspect of severe AS interfering with cerebral autoregulation is chronic cerebral hypoperfusion. Years after the maladaptive myocardial changes begin, the outflow obstruction leads to reduced stroke volume, resulting in loss of cerebral blood flow and the ability to attenuate blood pressure fluctuations (Carabello, 2013). Patients with smaller baseline aortic valve areas showed reduced phase improvements after SAVR at low frequencies. This means that the severity of the disease has a negative effect on the post‐surgical dCA outcomes, even if they are not relevant when compared to baseline assessments. Since stroke volume is dependent on the left ventricular outflow tract area, a smaller aortic valve area may be accountable for aggravating cerebral blood flow (Poh et al., 2008). Evidence on heart transplantation showed improvement in dCA in patients with CHF (Gruhn et al., 2001)^,^ suggesting that the CBF in patients with AS depends not only on dCA but also on the restoration of cardiac output (CO). Several past works have shown a conflicting contribution of acute changes in CO in determining CBF, but none has studied the role of the chronic reduction in CO in cerebral haemodynamics (Deegan et al., 2010). Although the duration of AS was not addressed in our analysis, Meng et al. proposed that long‐term impairment of CO upregulates the renin–angiotensin–aldosterone system (RAAS). The RAAS activation not only contributes to cardiac remodelling but also increased cerebral vessel resistance (Meng et al., 2015). The downregulation of RAAS, in particular the inhibition of angiotensin‐II receptors, was shown to augment cerebral autoregulation and to delay cerebral blood flow decline after middle cerebral artery occlusions (Yamakawa et al., 2003).

Changes in gain at low and very low frequencies and in phase at low frequencies were not statistically significant. While the chronology of the compensatory mechanisms in brain haemodynamics triggered by AS is unknown, our patients were evaluated 6 months after SAVR, so long‐term effects on dCA and VR might yet be reverting at the moment of the post‐operative visit. In contrast to dCA, baseline brain vasoreactivity to CO_2_ was impaired in AS patients, which accords with previous studies where a lower breath‐holding index was found in this subgroup (Scuric et al., 2013). When ejection fraction is reduced, Georgiadis (2000) demonstrated a restriction in the recruitment of cerebrovascular reactive dilatory capacity.

Our study had advantages and limitations worth discussing. The inclusion of a healthy control group allowed us to compare the natural course of cerebral autoregulation in patients with severe aortic valve stenosis, which was a drawback stated in previous literature (Vlastra et al., 2021). We also provide unbiased results in terms of age and sex differences. However, we could not retrieve echocardiographic disease features from medical records for all patients and the small sample size may have impacted the statistical power of the baseline and pairwise comparisons. As older patients tend to exhibit other cardiovascular comorbidities that inevitably influence cerebral haemodynamics, finding the optimal sample for cerebral autoregulation studies is difficult. There were two patients with a history of stroke and atrial fibrillation that could potentially interfere with cerebrovascular control and haemodynamics independent of AS or SAVR. To address this issue, we conducted a sensitivity analysis excluding these patients from our sample and retrieved similar results regarding baseline dCA and CVR, as well as their changes after SAVR (Supporting information). We registered low coherence recordings in the very low‐frequency range that could potentially interfere with the linear input–output relationship implied in the transfer function analysis (Panerai et al., 2022). This was particularly evident when large time‐varying changes in arterial diameter led to adaptations in the haemodynamic resistance, thereby decreasing coherence. Moreover, a 2‐min interval may not be sufficient to achieve a steady state during the CO_2_ challenges (Carr et al., 2021), particularly in patients with severe aortic stenosis and concomitant heart failure. During the CO_2_ vasoreactivity assessment protocol, all instant physiological signals (CBFV, BP, capnography) were visually inspected to detect possible underperformance and the manoeuvre was repeated if this was the case.

## 5 CONCLUSION

Severe AS does not seem to impact cerebral autoregulation in the long term, but a decrease in brain VR to CO_2_ was found. The baseline aortic valve area may be used to identify patients with higher dCA post‐operative variations. SAVR does not improve cerebral haemodynamics, possibly due to cardiac remodelling and altered brain vascular resistance. Cerebral haemodynamic changes in AS showed a close relationship with stroke volume, so further studies addressing this indicator are warranted.

## AUTHOR CONTRIBUTIONS

Andreia Costa and Pedro Castro conceived the study design; Andreia Costa, Ana Luísa Rocha, Juliana Ferreira, Elson Salgueiro, and Gilberto Pereira collected the data; Tiago Pedro analysed the data; Tiago Pedro, Andreia Costa, and Pedro Castro interpreted the results; Tiago Pedro prepared the figures; Tiago Pedro and Pedro Castro drafted the manuscript; Tiago Pedro and Pedro Castro edited and revised the manuscript. All authors have read and approved the final version of this manuscript and agree to be accountable for all aspects of the work in ensuring that questions related to the accuracy or integrity of any part of the work are appropriately investigated and resolved. All persons designated as authors qualify for authorship, and all those who qualify for authorship are listed.

## CONFLICT OF INTEREST

The authors have no relevant conflicts of interest to declare.

## FUNDING INFORMATION

The authors did not receive financial support from any organization for the submitted work.

## Supporting information

Table S1. Results for baseline dynamic cerebral autoregulation comparison between controls and pre‐SAVR AS patients.
Table S2. Results for pairwise comparisons between pre‐SAVR and post‐SAVR AS patients regarding cerebral autoregulation parameters.

Statistical Summary Document

## Data Availability

The data that support the findings of this study are available from the corresponding author upon reasonable request.
