# Supplementary material for: Changes in cerebral autoregulation and vasoreactivity after surgical aortic valve replacement: a prospective study
Source: Exp Physiol. 2022 Nov 20;108(1):103–10. doi: 10.1113/EP090502 (PMC10103757; doi:10.1113/EP090502)
Supplement: Supplementary file 1 — Table S1. Results for baseline dynamic cerebral autoregulation comparison between controls and pre‐SAVR AS patients. Table S2. Results for pairwise comparisons between pre‐SAVR and post‐SAVR AS patients regarding cerebral autoregulation parameters. [file EPH-108-103-s001.docx]

**Supplemental material 1**

| **TABLE 1 \| Results for baseline dynamic Cerebral Autoregulation comparison between controls and pre-SAVR AS patients.**  **Test Statistics^a^** | | | | | | | | | | | | | |
| --- | --- | --- | --- | --- | --- | --- | --- | --- | --- | --- | --- | --- | --- |
|  | mCO_VLF | mCO_LF | mCO_HF | mGAIN_VLF | mGAIN_LF | mGAIN_HF | mphase_VLF | mphase_LF | mphase_HF | BPm | HRm | ETCOm | Tsk1VMR |
| Mann-Whitney U | 128.000 | 119.000 | 136.000 | 117.000 | 145.000 | 123.000 | 108.000 | 139.000 | 147.000 | 52.000 | 146.000 | 90.000 | 70.500 |
| Wilcoxon W | 338.000 | 329.000 | 346.000 | 237.000 | 265.000 | 333.000 | 228.000 | 349.000 | 357.000 | 262.000 | 266.000 | 280.000 | 260.500 |
| Z | -.733 | -1.033 | -.467 | -1.100 | -.167 | -.900 | -1.197 | -.367 | -.100 | -3.267 | -.133 | -1.566 | -2.498 |
| Asymp. Sig. (2-tailed) | .463 | .301 | .641 | .271 | .868 | .368 | .231 | .714 | .920 | .001 | .894 | .117 | .012 |
| Exact Sig. [2*(1-tailed Sig.)] | .479^b^ | .314^b^ | .657^b^ | .283^b^ | .882^b^ | .382^b^ | .242^b^ | .730^b^ | .934^b^ | **.001^b^** | .908^b^ | .123^b^ | **.011^b^** |
| a. Grouping Variable: Group1teste2controlo | | | | | | | | | | | | | |
| b. Not corrected for ties. | | | | | | | | | | | | | |

| **TABLE 2 \| Results for pairwise comparisons between pre-SAVR and post-SAVR AS patients regarding Cerebral Autoregulation parameters.**  **Test Statistics^a^** | | | | | | | | | | | | | |
| --- | --- | --- | --- | --- | --- | --- | --- | --- | --- | --- | --- | --- | --- |
|  | mCO_VLF_R - mCO_VLF | mCO_LF_r - mCO_LF | mCO_hF_r - mCO_HF | mGAIN_VLF_r - mGAIN_VLF | mGAIN_LF_R - mGAIN_LF | mGAIN_HF_R - mGAIN_HF | mphase_VLF_R - mphase_VLF | mphase_LF_R - mphase_LF | mphase_HF_R - mphase_HF | BPm_R - BPm | HRm_R - HRm | ETCOm_R - ETCOm | Tsk1VMR_R - Tsk1VMR |
| Z | -1.156^b^ | -.800^b^ | -1.867^b^ | -.622^c^ | -.267^c^ | -1.067^b^ | -.978^c^ | -.533^c^ | -.445^b^ | -2.134^b^ | .000^d^ | -.051^b^ | -.734^c^ |
| Asymp. Sig. (2-tailed) | .248 | .424 | .062 | .534 | .790 | .286 | .328 | .594 | .657 | **.033** | 1.000 | .959 | .463 |
| a. Wilcoxon Signed Ranks Test | | | | | | | | | | | | | |
| b. Based on negative ranks. | | | | | | | | | | | | | |
| c. Based on positive ranks. | | | | | | | | | | | | | |
| d. The sum of negative ranks equals the sum of positive ranks. | | | | | | | | | | | | | |
